# Teaching Hospitals

**Published:** 1918-07-20

**Authors:** Bertrand Dawson


### THE FUTURE OF THE MEDICAL PROFESSION.

### II.—TEACHING HOSPITALS.

By Sir Bertrand Dawson, K.C.V.O., C.B., M.D.

Being the Second Cavendish Lecture, delivered at the Royal Society of Medicine on July 11.

The teaching hospitals must henceforth be guarded and retained for their special objects. Their prime functions were education and research, not to serve the clinical needs of their district. Each such hospital should gather its patients from everywhere, and it would no doubt be fed from hospitals and clinics far and wide, as it belonged to the nation. It should be affiliated to a university or the Ministry of Education. Attached to it would be various institutes where medicine and social service would meet. Various special subjects would be directed by distinguished specialists who would be on the staff of the hospital. These institutes would give post-graduate training to doctors, and instruction to nurses and social workers.

Elections to the staff of a teaching hospital should not be exclusively in the hands either of the staff or the administration of the hospital. An academic council, selected by the university, should also have powers of recommendation, and the claims of all suitable candidates should be considered. The speaker considered that the size of the staff should be less rigid; because a physician or surgeon resigned it should not be thought a necessity forthwith to fill the vacancy; also, the distribution of beds definitely among the staff should cease, and the number of beds allotted to a resigning member should not necessarily descend to his successor. All members of the staff should commence with only a few beds, and the number could increase with the ability of any particular surgeon or physician. This was preferable to a man inheriting a clinic. There should always be beds available for collective investigation of problems arising from time to time. Team-work here also was essential for full results, as had been so well exemplified in the recent investigation into trench fever. One half the beds of a hospital he would allocate for the provision of each member of the staff with his minimum; the remainder would be available for strengthening clinics, and for collective investigations.

### Dangers of Whole-time Service.

It might be asked why medicine should not be placed under one professor, and surgery under another. Sir Bertrand's answer was that such would be a retrograde step: each of these subjects was too big to be compassed by the brain of one man. Moreover, under such a scheme there would be danger that thought would be cramped and stereotyped into one pattern; and if the individual happened to be unsuitable the whole department must become sterile for years at a time. He objected to a whole-time service on salary for teaching hospitals, as that would produce a narrow caste of academic men, who would be aloof from the problems and difficulties presented by disease in the world of action, disease which varied according to its environment. Still, a limited application of the whole-time principle to teaching hospitals he considered to be sound. For the first five years after his appointment every member of the staff should be a whole-time officer and be forbidden private practice. These years should be devoted by him to training, teaching, and research; and he would be saved from the struggle for scratch fees. During the next ten years a member of the teaching staff should give half his time to the service. When men arose whose tastes were entirely academic, let them remain whole-time workers at a salary. With the inevitable spread of institutional treatment consultants would more and more focus their private work in a single institution: this would compensate for the larger demand of the hospital on their time. To avoid staleness members of the staff should periodically be sent away for three to six months on study tours. The old-fashioned line of demarcation between the work of the physician and that of the surgeon should cease. Half of their beds should be common to both of them, for a large number of diseases had both surgical and medical aspects. Radiographers and laboratory workers should be brought into closer contact with the wards. Each member of the staff should have, besides his resident officer, two or more clinical assistants; these would be whole-time workers devoting themselves to work in their chief's wards, to teaching and research, at least one-third of their time being given to study and research work of their own. The salaries allotted should be proportioned to the time and energy devoted by each to the work of the hospital.

### Some State Aid Necessary.

No teaching hospital could provide the equipment and staff necessary without endowment from the State, and such State aid, on a small scale, was already in being. And, no doubt, the Health Ministry, if formed, would subsidise health clinics. Such a policy was quite compatible with the continuance of voluntary support combined with adequate central control. On the other hand, some of the great voluntary funds, like King Edward's Hospital Fund, maintained that narrow outlook which forbade the devotion of any part of their grant to investigation, though the results from the latter might be infinitely greater than those due to the treatment of the immediate needs of patients. It could still be said that in England teaching and research were starved. In European countries the State endowed them, in America a far-sighted millionaire did so, but help in England came from neither source.

The scheme he had outlined meant change, but not upheaval, and it preserved the principle that the medical student should be taught his profession by personal contact with the sick; and hence he was trained not only in knowledge but in sympathy, and could appreciate that great fact that the symptoms of disease were not constant in different individuals.

### Importance of Post-graduate Study.

There was need for more organisation of post-graduate study; doctors should be encouraged periodically to renew their contact with the schools, their salaries being continued during such "study leave." There was the more need for this organisation because after the war Great Britain and the Allied countries would be the goal of doctors from the Dominions and the United States who had previously been in the habit of visiting Germany and Austria. Similarly, large numbers of doctors in this country would cross to the States for a similar purpose.

Sir Bertrand proceeded to discuss the organisation we possess to secure the early diagnosis of disease, which was so supremely important. The profession was still too much occupied with the end-results. He gave a number of well-known examples, devoting special attention to "strain" in its various manifestations in our complex life.

With regard to sanatoria and spas, the proper conception of these was as fields for early and preventive treatment. On the outskirts of big cities there should be sanatoria, fully equipped, to which city men could resort while still maintaining their business activities. And each patient so treated would become a missionary to bring a health leaven into the community. What the Army could do in war surely civilians could do in peace. The medical profession could not provide the rich men, but it could and would supply the skilled advisers, and the organisation must be medical throughout. The spa presented the same idea as the sanatorium, on a larger scale, and with the difference that the patients rested from their avocations during the treatment. He recounted the climatic, scenic, geological, and other features which should be sought in spas, the particular waters found there being more or less subsidiary.

Attempts were being made by responsible bodies to guide the profession along the paths of sound thinking and effective action. The British Medical Association had devoted much ability and infinite pains to thinking out a scheme of constitution for a Ministry of Health. The Royal College of Physicians were forming a joint committee, which would be so enlarged as to make it specially representative of the academic side of medicine and surgery, and the work of this committee would in no way conflict with the work of the British Medical Association, but would, he hoped, be complemental to it. He believed that the profession had never before been faced with issues so momentous as now, and he hoped a large and comprehensive outlook would prevail, and strong and effective action be taken.

### Discussion.

The Chairman considered that the thanks of the whole profession were due to Sir Bertrand Dawson for the time and thought he had given to this very complicated question. He (Dr. Rice-Oxley) advocated a greater participation by medical men in municipal affairs.

Major Henry Dutch thought Sir Bertrand Dawson had formulated his views on the exigencies of the present military service; his local hospital was a millennium ideal. Evidently, however, the lecturer had got the good of the profession, and especially of the junior practitioner, at heart. But if the latter were allowed, early in his career, to undertake operations for strangulated hernia, subclavian artery ligature, and Gasserian ganglion conditions, the undertaker and the monumental mason would be unduly busy. All schemes for betterment in this way would be ineffectual unless and until the whole profession combined to bring them into being.

Sir John Broadbent, M.D., entered into a detailed critique of the lecture. He said he thought a complete State Service would be the ruination of medicine: the doctor, under that, would receive a salary and would be without the stimulus to do better work; and he would receive no promotion unless a senior died or resigned. The compromise suggested by Sir Bertrand Dawson possessed several merits. The scientific interests of the doctors would not be allowed to lapse, as they would be in touch with bacteriologists, pathologists, and consultants who would give them advice on their cases. The drawback would be that every doctor in a large district could not be put on the Centre; and if some six or eight out of forty were put on, the remainder would feel slighted. And it would require something only a little short of military discipline to compel patients to attend a given Centre at a fixed time, though the scheme set out was an admirable one if it could be carried through. At present it was very difficult for the medical practitioner to bring his patients into close touch with the various special departments of a hospital. He would like to see attached to voluntary hospitals a series of paying wards, to which patients could be admitted who would pay according to their means; but it would be, in his opinion, a great mistake to place hospitals under Government control. Voluntary committees did an enormous amount of valuable work; they took a great interest in the institutions, patients were visited by them, and every care was taken to render them comfortable. With regard to putting them under a university, the University of London was only an abstract term: the authorities there laid down a lot of restrictions in regard to medical teaching, but they did not give any support or grant-in-aid to teaching, a disastrous state of things. He agreed that preventive and curative medicine should go hand-in-hand, but it was too much to expect the ordinary medical man to be well up in such subjects as bacteriology, etc., which entered so largely into preventive medicine. He thought Sir Bertrand Dawson had done a very great service in opening up this question in the way he had.

### The Practitioner and Public Health.

Dr. E. H. M. Stancomb (Southampton) said much depended on how the profession was organised. The vast waste of human life, and the premature disease and decay observable constituted a continual stimulus to the medical profession. Often the general practitioner had forgotten his preventive medicine. Patients often returned from a sanatorium robust and vastly improved, but resumption of the old life led to a relapse. There must be less overcrowding in houses, and the practitioner must become more and more a pillar of public health; he must be indoctrinated with local hygiene. He doubted whether strain was produced by work; it was rather due to the conditions of life generally. He laid great stress on suggestive therapeutics, and would like psychology to be a compulsory subject in the curriculum. The profession was at present struggling under conditions of sordid inadequacy of means and equipment.

Other speakers followed, but few points were made. Indeed, the whole discussion was most disappointing and may be taken as evidence of the relatively small amount of interest exhibited by the general body of members of the profession in the scheme unfolded by Sir Bertrand Dawson. Sir Bertrand in his reply insisted that it was not necessary to put up everywhere the kind of hospital he had suggested. His scheme must be evolved as required by local circumstances. Sir Bertrand held that the real question for the profession was to consider whether they proposed to lead or to be dragged. He suggested that the machinery for ascertaining the views of the medical profession was the British Medical Association, but as that body represented at the outside only about a third of the qualified and working members of the profession, the suggestion did not offer much encouragement to those who are genuinely anxious to secure a move on in this matter. To be quite frank, the Cavendish Lecture this year cannot be regarded as a brilliant success. It causes ardent spirits to feel that more life must be introduced into these lectures in the future or they may gradually disappear through inanition. We had no space to publish the first part of the lecture, which will, however, be found in full in the *British Medical Journal* of July 13.

